# Forecast of Outpatient Visits to a Tertiary Eyecare Network in India Using the EyeSmart Electronic Medical Record System

**DOI:** 10.3390/healthcare9060749

**Published:** 2021-06-18

**Authors:** Gumpili Sai Prashanthi, Nareen Molugu, Priyanka Kammari, Ranganath Vadapalli, Anthony Vipin Das

**Affiliations:** 1 Department of EyeSmart EMR & AEye, L. V. Prasad Eye Institute, Hyderabad 500034, India; saiprashanthi.g@lvpei.org (G.S.P.); priyankakammari19@gmail.com (P.K.); ranganath@lvpei.org (R.V.); 2 Indian Health Outcomes, Public Health and Economics Research Center, L. V. Prasad Eye Institute, Hyderabad 500034, India; 3 LVPEI Center for Innovation, L.V. Prasad Eye Institute, Banjara Hills, Hyderabad 500034, India; nareenmolugu@gmail.com

**Keywords:** forecasting, electronic health records, health resources, patient flow, SARIMA

## Abstract

India is home to 1.3 billion people. The geography and the magnitude of the population present unique challenges in the delivery of healthcare services. The implementation of electronic health records and tools for conducting predictive modeling enables opportunities to explore time series data like patient inflow to the hospital. This study aims to analyze expected outpatient visits to the tertiary eyecare network in India using datasets from a domestically developed electronic medical record system (eyeSmart™) implemented across a large multitier ophthalmology network in India. Demographic information of 3,384,157 patient visits was obtained from eyeSmart EMR from August 2010 to December 2017 across the L.V. Prasad Eye Institute network. Age, gender, date of visit and time status of the patients were selected for analysis. The datapoints for each parameter from the patient visits were modeled using the seasonal autoregressive integrated moving average (SARIMA) modeling. SARIMA (0,0,1)(0,1,7)_7_ provided the best fit for predicting total outpatient visits. This study describes the prediction method of forecasting outpatient visits to a large eyecare network in India. The results of our model hold the potential to be used to support the decisions of resource planning in the delivery of eyecare services to patients.

## 1. Introduction

The geography and the magnitude of the population in India present unique challenges in the delivery of healthcare services. Digitization of patient records brings with it the benefits of real-time access to information and enables a better strategy for enhancing future care. Various studies have predicted patterns in patient care such as patient flow for outpatient consultation [1], discharge prioritization for inpatients [2], readmission rates in hospitals [3,4] and adverse drug reactions [5]. Ramos et al. [1] described a predictive model which calculated the patient flow based on geospatial variables in a medium-sized Spanish city. Patients who had a higher socioeconomic status and those in the age groups of 25–34 and 55–65 years paid visits to health centres less often. Monday and Friday were the days of the greatest and lowest demand, respectively. February had the highest influx of patients on a monthly basis. There were significantly more patient visits during days with poor air quality and high relative humidity. Barnes et al. [2] presented a model which used supervised machine learning methods to predict the total number of discharges in a day for an inpatient medical unit. McLaren et al. [3] described a model which predicted the readmission rates among heart failure patients based on their prior admission status. They observed that the risk of readmission significantly increases with the increase in the number of prior admissions after adjusting for the related clinical variables. Zhao et al. [5] proposed a predictive model to identify adverse events due to drugs and further presented solutions to few of the challenges it presented.

The use of statistical modeling to predict healthcare outcomes is breaking new ground in the field of biomedical informatics and data science. The implementation of electronic health records (EHR) and tools for conducting predictive modeling enables vast opportunities to explore time series data like patient inflow to the hospital. Historical traits are assumed to manifest themselves in the future in time series models [6]. Autoregressive integrated moving average (ARIMA) is a univariate stochastic model that uses probability and statistics for forecasting purposes. Seasonal ARIMA (SARIMA) modeling approach, a variant of ARIMA, is used in time series that have a tendency of showing a periodic behavior after certain time intervals [7]. Efficient management of eyecare services can be enhanced with predictive forecasts of future eyecare demand and resource availability in real time. In the present analysis using a SARIMA model, we aimed to evaluate the expected outpatient visits to a large tertiary eyecare network in India. We present a predictive model to forecast the presentation of patients that can assist in better resource allocation for an efficient delivery of eyecare services.

## 2. Materials and Methods

EyeSmart Electronic Medical Record (EMR) (eyeSmart™) is an electronic medical record system developed in-house at the L.V. Prasad Eye Institute, India. The project which started in August 2010 has successfully completed digitization of the entire LVPEI network which comprises centers which span four states of India. On the average, 4500 patients visit the network on a given day. The EMR system allows structured documentation of demographic and clinical information which are stored in the database of the respective centers. The study protocols and procedures adhered to the tenets of the declaration of Helsinki [8]. The data were made anonymous prior to access by the authors.

### Data Preparation

Demographic information of 3,384,157 patient visits was obtained from eyeSmart EMR from August 2010 to December 2017 across the LVPEI network. Data of the four tertiary centers, namely, Hyderabad campus (KAR), Vishakhapatnam campus (GMRV), Bhubaneswar campus (MTC) and Vijayawada campus (KVC) were considered for analysis. The length of time series data of patient visits was 1250 days for the KAR, 1809 days for the GMRV, 1986 days for the MTC and 2793 days for the KVC. The parameters of age, gender, date of visit and time status of the patients were selected for analysis. The subset of patients 0–1 years of age and all the patients who presented on time for the appointment were excluded from the analysis due to sparse data. The time status of the patient at presentation was defined as “before time” or “delayed” with respect to their time of appointment. The datapoints in each parameter from the patient visits were modeled using a train—test split, with a ratio of 80:20. This split was formed for the development and validation of the predictive model. The data for training and testing the model were selected from the KVC with 481,262 outpatient visits. The training and testing sets were separated by time. The training data consisted of 2241 days. The predictive model was tested by a one-week prediction of the parameters for 52 weeks. The model was trained on the data which were recorded before the date and validated on the data from a week ahead from the date.

## 3. Results

The following sequential steps were followed for SARIMA modeling and forecasting.

### 3.1. Exploratory Data Analysis and Data Preprocessing

#### 3.1.1. Data Visualization

**Figure 1:**
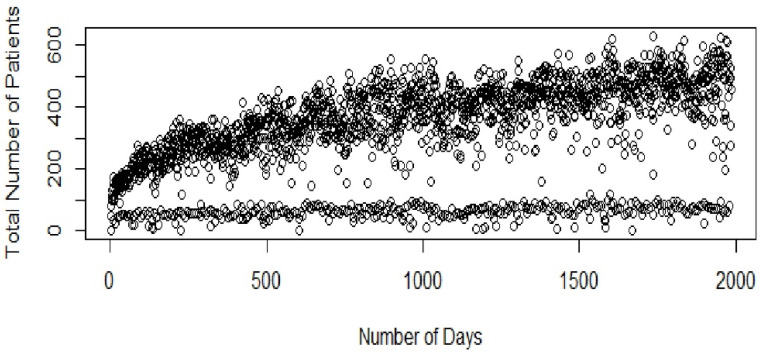
Total patient inflow (KVC).

The daily outpatient visits show an overall increasing trend (Figure 1). A significant number of patients visited the center on most of the days except for few days like sundays where there was a dip in the total number of patients who visited the center.

**Figure 2:**
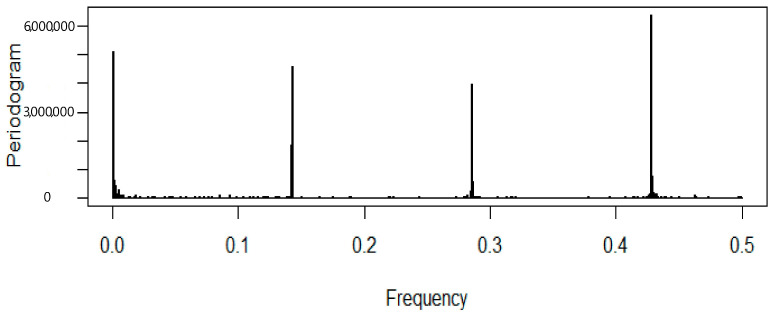
Periodogram of the total patients (KVC).

The periodogram of the total number of patients suggests weekly seasonality (Figure 2). The periodogram shows spikes at frequencies of 0.14, 0.28, 0.42, etc., confirming seasonality with a time period of seven days.

#### 3.1.2. Stationarity Check

**Figure 3:**
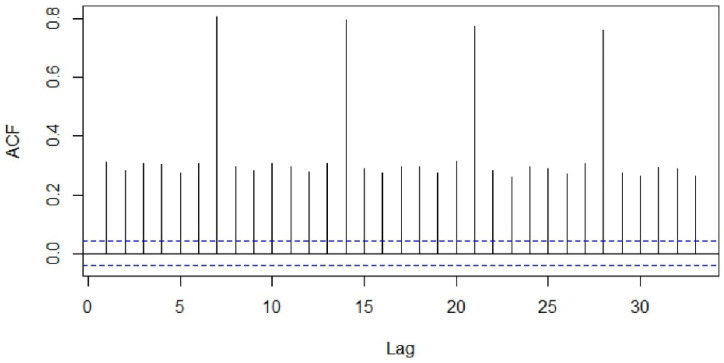
Autocorrelation function of the total number of patients.

**Figure 4:**
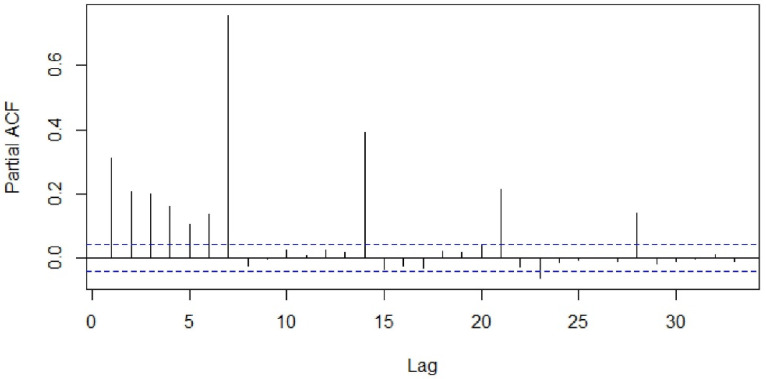
Partial autocorrelation function of the total number of patients.

Visualization tools such as the sample autocorrelation function (ACF) and sample partial autocorrelation functions (PACF) were used to examine stationarity of the series (Figure 3 and Figure 4). The Kwiatkowski–Philips–Schmidt– Shin (KPSS) test at the 0.05 significance level (α = 0.05) was used to understand the type of non-stationarity in the data and suggested the necessary transformations. From the KPSS test, it was observed that the series which is differenced with the lag of 7 was trend-stationary. The autocorrelation plot (Figure 3) showed spikes at lag 7 and its multiples and the partial autocorrelation plot (Figure 4) demonstrated an initial significant spike with a rapid decline at multiples of lag 7, both indicating a seasonal moving average term. Similar observations were in other categories as well; hence, similar procedures were followed to model other time-series data.

### 3.2. Model Identification and Diagnostic Checking

**Figure 5:**
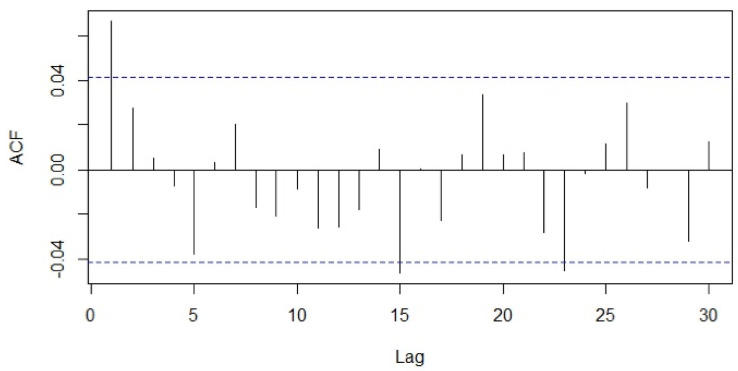
Autocorrelation function of residuals of ARIMA (0,0,0)(0,1,7).

**Figure 6:**
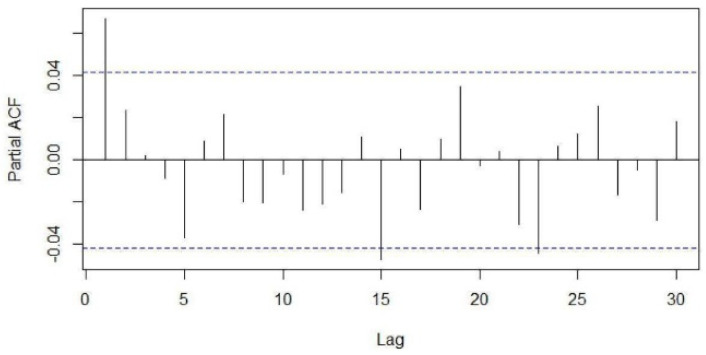
Partial autocorrelation function of residuals of ARIMA (0,0,0)(0,1,7).

The type (seasonal or non-seasonal) and order (p, q, P, Q) of model parameters were identified on the basis of the ACF, PACF diagrams and Akaike information criterion (AIC) values. Different seasonal moving average (MA) models as suggested by the ACF and PACF plots were applied to our differenced data and the proximity of the model residuals to white noise was observed. The ACF and PACF of residuals of the ARIMA (0,0,0)(0,1,7)_7_ applied to the time-series data were closest to the white noise (Figure 5 and Figure 6). However, the ACF and PACF values observed at lag 1 suggested additional AR (autoregressive) or MA terms in our present model.

**Figure 7:**
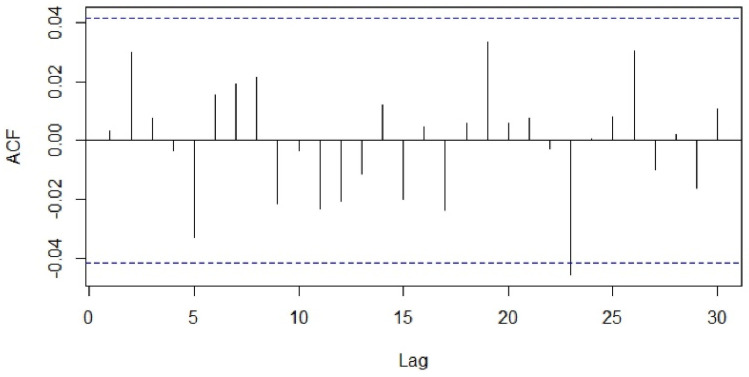
Autocorrelation function of residuals of ARIMA (0,0,1)(0,1,7).

**Figure 8:**
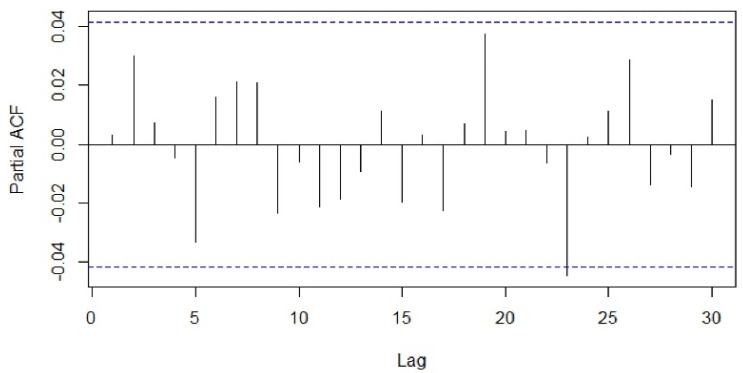
Partial autocorrelation function of residuals of ARIMA (0,0,1)(0,1,7).

ARIMA (0,0,1)(0,1,7)_7_ was found to be the best model for the total patient footfall data with an estimated sigma square of 1864, log likelihood of −11,548.23 and AIC value of 23,114.46. The majority of values of the ACF and PACF plots were within the confidence intervals which resemble the signature plots for white noise (Figure 7 and Figure 8). The selection of the model for the respective parameters based on the AIC values and parsimony criteria is detailed in Table 1.

### 3.3. Evaluation Protocol

Our training and testing sets were separated by time. The model was trained using data from the past and applied to the future data. The training data consisted of 1986 days from 2 June 2013 to 7 October 2017. The testing data consisted of 364 days from 8 October 2017 to 7 October 2018.

### 3.4. Measuring Forecast Performance

We compared the forecasts obtained using our proposed approach with the actual recorded values from the EMR on the measures of the root-mean-square error (RMSE), mean absolute error (MAE), mean absolute percentage error (MAPE) and symmetric mean absolute percentage error (SMAPE).

The modeling process found the following SARIMA, (0,0,1)(0,1,7)_7_, provided the best fit for predicting total outpatient visits, generating the median of 37.56, 48.01 and 16.82% for MAE, RMSE, MAPE, respectively, in the validation dataset. Table 2 shows median error values of the predictive model.

**Figure 9:**
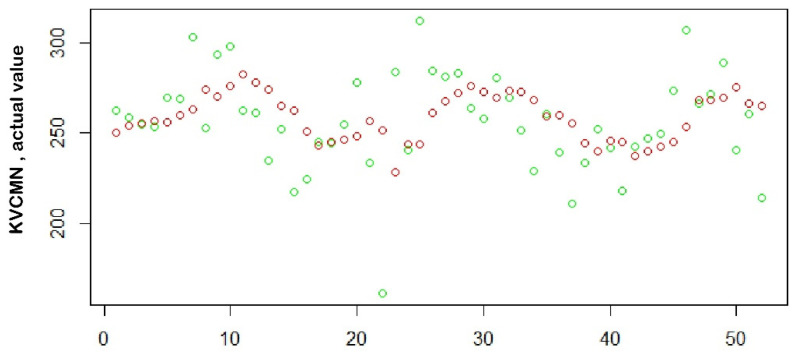
Comparison of actual (green) versus predicted (red) values.

Different SARIMA models (Table 1) were used to make predictions of total outpatient visits and associated parameters for the week ahead. The prediction model was run for 52 weeks, spanning an entire year from 6 May 2017 to 6 May 2018. We used the SMAPE as the performance metric to account for the days where the actual value was zero. The comparison of total outpatient visits between the actual and predicted values for the KVC campus is shown in Figure 9. The number of weeks where the prediction was within the 20% margin of the SMAPE is detailed in Table 3.

**Figure 10:**
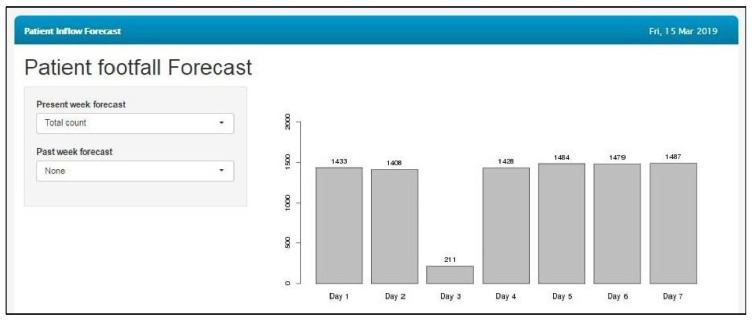
Prediction of the total patient count for the next seven days.

Figure 10 shows the prediction of the total patient footfall for the next seven days; an option to visualize a one-week-ahead forecast of variables such as the total count, male count, female count, pediatric count, adult count, delayed patient count and count of patients who are before the appointed time is given in the dropdown list. As a part of the ongoing validation of the model with the actual footfalls in the hospital, actual vs. predicted values of the previous seven days were showcased to assess the accuracy of the prediction. The forecasting model was integrated into the eyeSmart EMR system for the administrative staff.

## 4. Discussion

### 4.1. Principal Results

This study described the methodology of predicting outpatient visits in a large eyecare network in India. The parameters of age, gender and time status at presentation were also predicted to gain better insights to provide efficient eyecare services. The modeling process found SARIMA (0,0,1)(0,1,7)_7_ to provide the best fit for forecasting total outpatient inflow. The MAPE of 16.82% was observed for the predictive model which forecasted the total outpatient inflow.

### 4.2. Comparison with Prior Works

Ramos et al. [1] predicted the expected outpatient visits to primary health centers in a medium-sized city in Spain. The study used multiple regression analysis and showed an improvement in error values on the addition of geospatial variables such as temperature, air quality, relative humidity and economic status. Further, Luo et al. [9] described a model which was used for forecasting outpatient visits daily about a week ahead from the data which contained one year of daily visits of outpatients to a large hospital in Chengudu. The combinatorial model which combined SARIMA and simple exponential smoothening (SES) was used in this study. The MAPE values of only the SARIMA model for respiratory and endocrine outpatient visits was 15.26% and 11.77%, respectively for a seven-day forecast horizon. In addition, Calegari et al. [10] forecasted daily visits to a tertiary care teaching hospital in Brazil using different mathematical models. Model accuracy was evaluated using the MAPE. Their MAPE at seven days was comparable, at 12.01%, using the SARIMA model. Huang et al. [11] used a hybrid prediction model which combined ARIMA and filtering to accurately forecast the demand for medical services in the medium as well as short term. Another retrospective study in a medical center in Taiwan forecasted emergency visits using time series analysis. They identified that ARIMA (0,0,1) was the best fit and yielded the MAPE of 8.91% [12]. A group in China conducted an observational study which analyzed patient’s visits across 10 years at an eyecare facility [13]. They observed a continuous increase in patient visits over the years leading to an increased demand and hence more burden on the eyecare system.

Numerous studies have been undertaken to forecast new admission inpatients, outpatient visits and visits to blood sampling rooms and emergency departments [14,15,16,17,18]. Predictive models can be used to support the decisions regarding the allocation of resources and can significantly reduce the burden on healthcare. In a country of 1.3 billion, data science holds a potential for predictive models to be applied to healthcare delivery. To the best of our knowledge, there is no study currently in literature describing the use of data modeling in predicting outpatient visits in eyecare in India.

### 4.3. Limitations

The model used in this study is used only for short-term forecasting. Apart from the time series data used for prediction, many other influence factors such as preference of a particular doctor by the patient and supply of outpatient resource will also have a significant impact on the visits of the patients. The model can be made more efficient by taking these features into consideration.

## 5. Conclusions

In conclusion, this study contributes to the exploration of the prediction method to forecast outpatient visits in India’s largest eyecare centers. Our study confirms that the daily patient inflow follows seasonal weekly patterns. Further, it shows that SARIMA models can be implemented to accurately predict forecasts of highly complex patient inflow data, mainly in the short term. This model is feasible in terms of implementation and computation of prediction for a week ahead, yielding small mean (0.02) and lower standard deviation (17.34) of the residuals. The current prediction model is integrated into the eyeSmart EMR system across the four tertiary centers in the LVPEI network to validate outpatient visits predictions in the real-life scenario. The results of our model hold a potential to be used to support the decisions of resource planning in the delivery of eyecare services to the patients.

Future work mainly involves building models which can forecast outpatient visits in the middle and long term. The inclusion of geospatial variables like temperature, air quality and relative humidity can also increase the prediction accuracy of the model. Our future work will involve addition of these variables to the model to increase prediction accuracy.

## Figures and Tables

**Table 1:** AIC values of possible SARIMA models.

Parameter	(0,0,0)(0,1,7)_7_	(0,0,1)(0,1,7)_7_	(1,0,0)(0,1,7)_7_	(1,0,1)(0,1,7)_7_
Male	20,507.11	20,506.7	20,506.8	20,508.45
Female	20,002.47	19,996.45	19,996.72	19,998.37
Pediatric	16,727.46	16,729.34	16,729.33	16,726.48
Adult	20,513.51	20,515.45	20,515.46	20,517.45
Elderly	19,066.63	19,051.54	19,049.88	19,049.98
Before time	21,557.36	21,556.42	21,556.58	21,558.15
Delayed	19,089.98	19,083.4	19,083.14	19,084.94
Total	23,117.58	23,114.46	23,114.66	23,116.3

**Table 2:** Median error values of the predictive model.

Parameter	Mean Absolute Error	Root-Mean-Square Error	Mean Absolute Percentage Error
Male	22.98	28.36	22.99%
Female	17.53	24.58	17.03%
Pediatric	11.84	15.07	62.75%
Adult	19.97	26.62	18.36%
Elderly	16.97	21.91	23.79%
Before time	30.16	36.72	44.17%
Delayed	22.36	27.10	18.56%
Total	37.56	48.01	16.82%

**Table 3:** Performance measurement of the model at 52 weeks (KVC).

Parameter	No. of Weeks (Error < 20%)
Male	51 (98.07%)
Female	50 (96.15%)
Delayed	51 (98.07%)
Before time	44 (84.61%)
Pediatric	37 (71.15%)
Adult	51 (98.07%)
Elderly	51 (98.07%)
Total	51 (98.07%)

## Data Availability

Data sharing not applicable.
